# Unusual Presentation of Wünderlich Syndrome

**DOI:** 10.31486/toj.21.0120

**Published:** 2022

**Authors:** Luis R. García-Chairez, Fred A. Montelongo-Rodríguez, Ilse A. Moreno-Arquieta, Max Molina Ayala, Adrián Gutierrez-González

**Affiliations:** ^1^ Department of Urology, Hospital Universitario Dr. José Eleuterio González, Universidad Autónoma de Nuevo León, Monterrey, Nuevo León, México; ^2^ Medical School Universidad Autónoma de Nuevo León, Monterrey, Nuevo León, México; ^3^ Department of Pathology, Hospital Universitario Dr. José Eleuterio González, Universidad Autónoma de Nuevo León, Monterrey, Nuevo León, México

**Keywords:** *Hemorrhage*, *kidney*, *nephrectomy*, *pyelonephritis*

## Abstract

**Background:** Wünderlich syndrome is a rare but important condition because it involves a sudden blood collection in the renal fossa that can cause hemodynamic instability.

**Case Report:** A 38-year-old female with a history of type 2 diabetes mellitus and hypertension with poor adherence to treatment presented to the emergency department with abdominal pain of 2 weeks’ duration accompanied by irritative lower urinary symptoms. Abdominal computed tomography (CT) scan showed bilateral pyelonephritis and an abscess in the lower pole of the right kidney. A second CT scan, performed because of the patient's abrupt decrease in hemoglobin and hematocrit, showed active bleeding secondary to the infectious process in the right kidney. The patient was hemodynamically unstable, so a nephrectomy was performed.

**Conclusion:** Wünderlich syndrome is a spontaneous renal hemorrhage, in most cases attributed to a tumorous etiology and rarely of infectious origin. The clinical picture is varied but can present with the Lenk triad of acute onset flank pain, flank mass, and hypovolemic shock. It is diagnosed principally via an imaging study such as abdominal CT scan. Treatment is conservative in principle, but urgent surgical intervention is sometimes necessary depending on the clinical situation of the patient.

## INTRODUCTION

Spontaneous renal hemorrhage of nontraumatic etiology, or Wünderlich syndrome, is a rare but significant condition that leads to a sudden blood collection in the renal fossa. Most authors agree the principal etiology of this hemorrhage is tumors (65%), followed by vascular pathology (20%-30%); infectious causes (12%); and other etiologies classified as miscellaneous because of their variety, including cystic renal diseases, nephrosclerosis, and preeclampsia (12.7%).^1-4^ Symptoms can be varied and nonspecific, so a high clinical suspicion is imperative for the proper management of patients with spontaneous renal hemorrhage. Also, the importance of identifying Wünderlich syndrome lies in its potential to cause hemodynamic instability, especially in patients with uncommon etiologies such as infectious disease.

We present the case of a 38-year-old female with an initial diagnosis of kidney abscess that became Wünderlich syndrome, changing our initial management with antibiotics to surgical intervention.

## CASE REPORT

A 38-year-old female with a history of type 2 diabetes mellitus and arterial hypertension under treatment with insulin but with poor medication adherence presented to the emergency department (ED) because of abdominal pain of 2 weeks’ duration accompanied by irritative lower urinary symptoms. She had previously self-medicated with gentamicin without clinical improvement and presented with fever, hyporexia, general malaise, and oral feeding intolerance. On her arrival in the ED, she had altered state of consciousness, hypotension, tachycardia, tachypnea, and generalized jaundice. Laboratory studies showed hemoglobin of 12.6 g/dL (reference range, 12.2-18.1 g/dL), hematocrit of 38.7% (reference range, 37.7%-53.7%), leukocytes of 38.8 K/μL (reference range, 4.0-11.0 K/μL), blood urea nitrogen of 157 mg/dL (reference range, 6-20 mg/dL), and creatinine of 8.0 mg/dL (reference range, 0.8-1.2 mg/dL).

**Figure 1.**
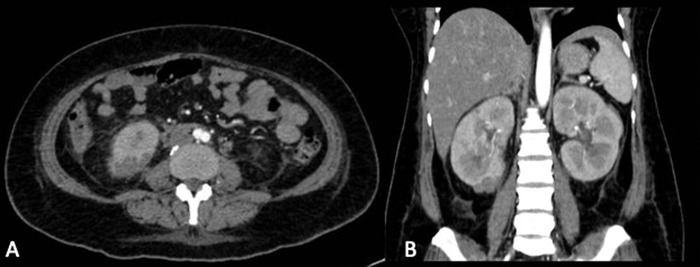
Abdominal-pelvic (A) transverse and (B) coronal computed tomography with contrast shows an abscess measuring 2.6 × 1.6 × 1.8 cm with a volume of 3.89 cc in the lower pole of the right kidney.

Abdominal ultrasound of the kidneys suggested a renal abscess. Abdominal computed tomography (CT) with contrast showed bilateral pyelonephritis with a defect in the parenchyma of the lower pole of the right kidney measuring 2.6 × 1.6 × 1.8 cm with a volume of 3.89 cc and 40 HU that supported the diagnosis of right renal abscess (Figure 1). The patient also had multiple lesions in the left kidney, the dimensions of the largest being 0.6 × 0.8 × 2.9 cm with a volume of 0.72 cc.

**Figure 2.**
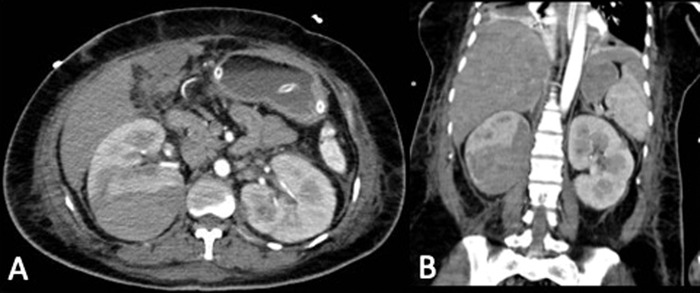
Abdominal-pelvic (A) transverse and (B) coronal computed tomography with contrast shows a perirenal hematoma with dimensions of 10 × 7.3 × 5.4 cm and a volume of 205 cc in the posterior region of the right kidney.

Because of her hemodynamic status, the patient was admitted to the intensive care unit (ICU), and antibiotic therapy was initiated with imipenem at a dose of 500 mg every 12 hours and norepinephrine 16 mg in 125 mL of 5% glucose at 5 mL per hour. On her third day in the ICU, the patient had abrupt decreases in hemoglobin (10.0 g/dL to 4.6 g/dL) and hematocrit (30.1% to 13.1%). Repeat abdominal CT scan with contrast showed a perirenal hematoma in the right kidney measuring 10 × 7.3 × 5.4 cm with a volume of 205 cc (Figure 2).

The clinical diagnosis was Wünderlich syndrome or spontaneous renal hemorrhage secondary to the infectious process in the right kidney. Right nephrectomy was performed without complications.

**Figure 3.**
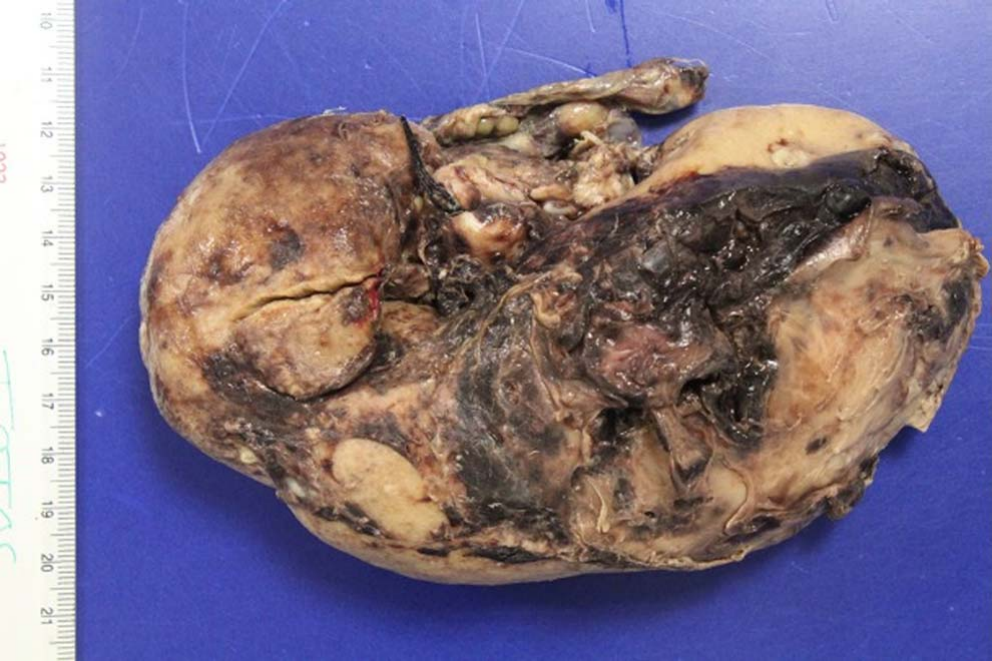
Macroscopic examination of the kidney revealed a hemorrhagic surface and laceration, with parenchymal rupture 0.6 cm deep, barely into the renal medulla.

**Figure 4.**
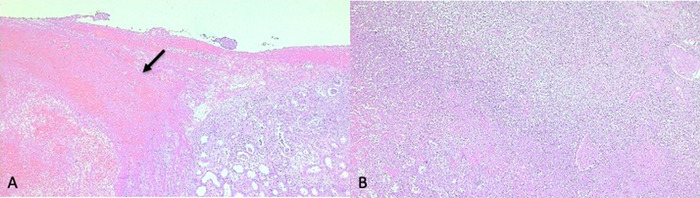
(A) Histologic section shows a vast hemorrhagic area with fibrin (arrow) next to renal parenchyma showing chronic pyelonephritis; however, (B) another section revealed acute inflammatory process beneath the hemorrhage. Therefore, acute pyelonephritis preceded the parenchymal rupture and hemorrhage.

Macroscopic study (Figure 3) described a kidney of 360 g measuring 14 × 5 × 7 × 5.2 cm, with a smooth external capsule and uneven consistency at the level of the upper pole where a violet-colored hematoma measuring 7 × 2.5 × 2 cm was identified, as well as evidence of multiple abscesses of a light yellow-greenish color and liquid consistency. The pathologic diagnosis was acute and chronic abscessed pyelonephritis with recent hemorrhage (Figure 4).

The patient had a favorable postoperative course, continuing with the imipenem and subsequent de-escalation of the antibiotic to levofloxacin 500 mg every 24 hours for 2 weeks based on the culture of the surgical sample, which was an *Escherichia coli*–producing beta-lactamase sensitive to quinolones and carbapenems. After 24 days of hospitalization, the patient had recovered from her surgery and the infectious process. She was discharged, and ambulatory follow-up was indicated.

## DISCUSSION

Bonet described spontaneous hemorrhage of renal cause for the first time in 1700, and Wünderlich defined it as “spontaneous apoplexy of the renal capsule” in 1856.^1,5^ In 1910, Coenen referred to this condition as “syndrome of Wünderlich.”^6^

The cause of Wünderlich syndrome is varied, with the most common etiology being malignant or benign tumors, as in the case of angiomyolipomas. Vascular diseases such as polyarteritis nodosa, renal artery aneurysms, and pseudoaneurysms are next in frequency.^2^ Uncommon causes of spontaneous renal hemorrhage are nephritis, hydronephrosis, polycystic diseases, and pathology of perirenal structures such as the adrenal glands; however, infectious etiology is even rarer.^1,7^ In a review of the literature by Cinman et al, the causes were tumors in 63% of cases (30% malignant tumors and 33% benign tumors), vascular disease in 25% of cases (the most frequent being polyarteritis nodosa), and infectious pathology in the minority of cases (12%).^3^

The form of manifestation varies depending on the amount of bleeding. The Lenk triad, defined as lumbar pain of sudden onset, rapid formation of palpable lumbar mass, and signs of hypovolemic shock, is manifested in only 20% of cases.^8^ However, Wünderlich syndrome has other insidious forms of clinical presentation as a consequence of moderate or severe bleeding and hemodynamic compensation.

The diagnosis can be difficult and is based initially on the diagnostic suspicion of blood loss, or on hemodynamic instability in some cases, and confirmed with imaging. For patients suspected of having Wünderlich syndrome, the evaluation should attempt to rule out traumatic antecedents, anticoagulant treatment, hemorrhagic diathesis, arteritis, tuberous sclerosis, and chronic hemodialysis.^9^ Our patient had no history of trauma; anticoagulant treatment; or any hemorrhagic, tumorous, or chronic diseases.

CT scan is the imaging modality of choice because it informs the degree of involvement of the kidney and adjacent structures, and, in most cases, allows a presumptive etiologic diagnosis to be established.^10,11^ CT is also the most widely used exploration study for the follow-up of patients who are conservatively managed.^1,9^ Renal arteriography is used when the etiologic suspicion is vascular, and embolizations can sometimes be therapeutic for lesions of vascular origin and for patients with benign lesions who have surgical contraindications.^6,9^

Treatment depends on the clinical situation of the patient. In hemodynamically stable patients, treatment is conservative, with abdominal CT scan used for diagnosis and follow-up.^9^ However, in unstable patients, urgent surgical intervention by nephrectomy may be indicated, which is not a first-line treatment because the surgery is associated with high morbidity and mortality.^12,13^ Our patient required emergency surgical intervention because of her hemodynamic instability and sudden decrease in hemoglobin and hematocrit, and she had a favorable postoperative outcome.

## CONCLUSION

Wünderlich syndrome is a rare condition but has the potential for vital compromise. Although the neoplastic origin is the most frequent, Wünderlich syndrome has many other less common etiologies, as was the case with our patient's infectious etiology. The clinical picture is varied but usually includes lumbar pain of sudden onset, rapid formation of palpable lumbar mass, and hypovolemic shock. CT is the diagnostic and follow-up method of choice. Treatment should be initially conservative, always considering the clinical and hemodynamic status of the patient. In the event of destabilization, emergency surgical intervention is necessary.

## Notes

*This article meets the Accreditation Council for Graduate Medical Education and the American Board of Medical Specialties Maintenance of Certification competencies for Patient Care and Medical Knowledge.*
